# Current News and Opinion

**Published:** 1884-02

**Authors:** 


### Current News and Opinion.

### THE TEETH OF THE FUTURE.

In an able address before the British Dental Association, at its meeting at Plymouth, its President, Mr. Spence Bate, F. R. S., has drawn attention to some remarkable features which it may be well for the profession to study. We have not the necessary space for the entire address, but while we acknowledge the eminence of the authority, we are not prepared to give credence to all his so-called facts, or agree with him in his deductions until he has been further substantiated by other observers.—*Editor.*

"In the teeth of the Esquimaux, the Red Indians and the natives of Ashantee, as well as those found in the ancient barrows of England, the so-called inter-globular spaces, seen so frequently in sections of modern teeth, appear not to exist; nor, indeed, are they to be detected in the dentition of the best developed structures of the modern European. Not only is the dentine getting deteriorated, but the enamel would seem likewise to be undergoing a modification, becoming too opaque. In addition to the histological changes, the external form and character of the teeth are sustaining an alteration. This seems to be in relation to an important feature in the history of their evolution. The tendency of the cranium to develop at the expense of the face and jaws is seen to occur as we ascend the scale of the vertebrated series of animals. Owing to this atrophy of the jaws, the proper space for the full play and development of the normal teeth would seem not to be available. At birth the bones are not sufficiently grown to receive the teeth in their normal arch; and, as in the human mouth, the pre-maxillary bones are firmly united a short time after birth, it follows that the posterior part of the jaw is the only place where growth can occur. Any delay in the development and consolidation of the symphisis must have the effect of contracting the space required for the teeth at this site In the course of vertebrate evolution there is a marked tendency for teeth to disappear. The lower vertebrates have four molars on each side in each jaw, the higher have three, while in man the number is reduced to two."

### THERAPEUTIC AGENTS FOR THE PROMOTION OF OSSEOUS DEVELOPMENT.

Dr. J. C. Thorowgood read a paper before the Odontological Society of Great Britain, on the above subject. He pointed out that the composition of the bones and the teeth was practically identical, the latter having only a larger proportion of inorganic matter. The analysis showed that a considerable quantity of mineral food was required for the nutrition of these tissues. The mere administration of the necessary lime-salts was, however, by no means the only thing to be considered in striving to improve osseous development; thus in rickets, with an evident deficiency of lime in the bones there was an elimination of from four to six times the normal amount of lime-salts in the urine, showing that the fault was in the process of elimination. For the dentist, the most serious condition in children was one of acid dyspepsia; the child's breath had a sour smell; tongue furred, with red papillae showing through; appetite often voracious, and bowels confined or irregular. To give a big-bellied, pale-faced child in this condition, phosphate of lime and iron, would only make him more uncomfortable; but give him alkaline aperients, regulate his diet, cutting off excess of sugar and starch, order exercise, salt-water baths, etc., and then administer the specific remedies indicated. Of these the most useful were the soluble hypophosphite of lime, and the chloride of calcium; either of these might be given in doses of two or three grains, in glycerine and water. The lactophosphate of lime was also a valuable remedy. Diet was most important. The child must be taught to eat slowly; brown bread and Scotch oatmeal would suit some children, and "seconds" flour was preferable to the "best white." By this line of treatment the child would be brought into a condition in which the dental surgeon could work on the decayed teeth with some prospect of his work remaining a lasting proof of his skill.—*Weekly Medical Review.*

---

### ZONULAR CATARACT WITH CHARACTERISTIC TEETH.

The characteristic teeth seen in zonular cataract resemble those seen in interstitial keratitis caused by hereditary syphilis. Zonular cataract has no connection with this disease. Associated with interstitial keratitis there are always peculiar physiognomical changes, but in quite half the cases these are of a very subtle nature, and difficult to describe. We see excellently formed teeth with no notches in the incisors, only a suspicious roundness at the cutting edges, and as often as not the bridge of the nose is well formed, but there is a peculiar softness and movability at the junction of the cartilage with the bone. We suspect eye or ear disease on seeing such patients approach us at a few yards. In zonular cataract there are no peculiarities about the features, and it is difficult to see what the connection may be of peculiar teeth with this abnormality in the eyes. In hereditary syphilis the teeth fall short of full development, in common often with many other structures, and eye diseases in relation with this occur up to late periods of growth. Zonular cataract is either congenital or develops only in very early life.—*Australian Medical Journal.*

---

### UNITS FOR MEASUREMENTS.

The metrical unit for length is the *meter;* the ten-millionth part of the distance from the earth's equator to the pole.

The unit of bulk is the *liter;* it is the cube of a decimeter side.

The unit of weight is the *gramme;* the weight of a cubic centimeter of distilled water at 40° Fahrenheit.

The unit of force is the *kilogram-meter;* the force required to raise one kilogram weight one meter high.

The unit of electric resistance is the *ohm;* it is the resistance which a current undergoes when passing through a column of mercury one meter long and one square millimeter in section, at the freezing point of water.

The unit of electro-motive force is the *volt*; it is the amount of electro-motive force produced by one Daniel cell.

The unit of electrical intensity is the *ampere;* it is the current produced by one volt through a resistance of one ohm.

The unit of quantity of current is the *calamb;* it is the quantity of electricity given by one ampere in one second.

### UNIVERSITY OF BUFFALO.

The spring term of the medical department of the University of Buffalo, will open immediately after the close of the winter term, and will continue eight weeks. Special courses of instruction have been arranged in most of the important departments, and the usual regular clinics will be held at the College, and at the General Hospital. The dissecting rooms will be open during the entire session, and the demonstrators will be in attendance daily. Arrangements have been made whereby students may attend the lectures of any special chair by paying the fee of the professor.

### FACULTY.

E. V. Stoddard, M. D., Materia Medica and Therapeutics.

M. D. Mann, M. D., Gynæcology.

F. W. Abbott, M. D., Otology.

A. M. Barker, M. D., Diseases of Children.

J. W. Keene, M. D., Obstetrics.

Frederick Peterson, M. D., Pathological Anatomy.

D. F. McPherson, M. D., Minor Surgery.

Eli H. Long, M. D., Special Therapeutics.

Charles Cary, M. D., General Anatomy.

R. A. Witthaus, M. D., Chemistry.

Roswell Park, M. D., General Surgery.

Lucien Howe, M. D., Ophthalmology.

Bernard Bartow, M. D., Orthopedic Surgery.

W. C. Barrett, M. D., Oral Diseases.

John H. Pryor, M. D., Physical Diagnosis.

Julius Pohlman, M. D., Physiology.

Frank A. Vandenburgh, Applied Chemistry.

---

### NAPHTHALINE AS AN ANTISEPTIC.

In consequence of the disagreeable action of iodoform on the organism, experiments have lately been made to discover an antiseptic without its objectionable properties. Prof. Fischer, of Strassburg, announces the discovery of the desired article in napthaline which can be used where iodoform was applied, without producing any constitutional action. It has been demonstrated that napthaline is an equally powerful antiseptic, but it has a less disagreeable odor. Its application produces a slight pain of short duration.—*Centralblatt fuer Chirurgie.*

### TRANSACTIONS (N. Y.) FOR 1882-83.

*Editor Independent Practitioner:*

Permit us through your journal to inform "whom it may concern" that the transactions of the Dental Society of the State of New York for 1882 and 1883 (one volume), have been published, and are ready for distribution.

The committee of publication has decided to issue them as follows, and will do so forthwith:

*First.* To district society members, through the secretaries of the several district societies.

*Second.* To permanent members, honorary members, masters of dental surgery not residents of this State, editors of dental journals, and State and national societies expressing their desire to exchange, through the Secretary of the Dental Society of the State of New York.

The errors to be found here and there throughout the volume are such as the reader will be able to correct at a glance, without any special directions from the committee.

Yours, etc.,

J. Edw. Line,

Chairman Com. of Pub.

Rochester, N. Y., Jan. 15th, 1884.

---

### RUSSIAN TEETH.

A Russian medical journal contains two reports on the frequency of caries in Russia. An examination of six hundred and fifty soldiers revealed the fact that two hundred and fifty-eight, almost forty per cent. suffered with carious teeth. In these cases the proportion of the diseased teeth in the upper and lower jaws was as two to three. The third molar was affected in about half the number of cases reported, while the incisor and canine teeth suffered least; the teeth of the right side had more vitality than those of the left. The other examination was made of the teeth of four hundred students in St. Petersburg. Of this number only twenty-eight per cent. were the happy possessors of healthy teeth. In two hundred and eightynine cases the molars were carious, the third molar in two hundred and forty-nine instances. It is stated by Prof. Skliffassorosky that eighty per cent. of the inhabitants of St. Petersburg have carious teeth. It seems that in Russia caries is just twice as prevalent in cities as in the country.—*Monatschrift des Vereins Deutscher Zahnkuenstler.*

---

### DISSOLUTION.

The firm of F. W. Leonard & Co. has been dissolved, and a new organization has been formed; The American Dental Manufacturing Company. Their place of business is upon Broadway, corner of 37th Street, New York. The announcement of the change will be found in our advertising pages, to which we refer readers for full particulars.

---

### THE AMERICAN MONTHLY MICROSCOPICAL JOURNAL.

During the past year this excellent journal has been published by S. E. Cassino & Co. of Boston. With the beginning of 1884, its editor, Romyn Hitchcock, F. R. M. S., resumes its full management and publication, and it will hereafter be regularly issued by him from Washington, D. C. It is but one dollar per year, and every microscopist should be a subscriber to it.

---

### CELLULOID DISKS.

The S. S. White Dental Manufacturing Co. are manufacturers of a wonderfully useful disk of celluloid, charged with corundum. They are exceedingly thin and flexible, and nicely adapted for polishing approximal fillings, especially in cases where disks of emery or sand-paper cannot be used on account of too much moisture, or lack of space.

C. E. F.

---

### REMOVED.

The New York Post Graduate Medical School has been compelled to move from its present quarters to more commodious ones, and will, on or about February 1st, 1884, occupy its new appartments in East 20th Street.

The new building is of sufficient proportions to admit of their combining hospital with school advantages.

FOR 1884.

Remittances for the INDEPENDENT PRACTITIONER come daily pouring in, and the treasurer hereby expresses his thanks for the prompt response to the slips enclosed in the December issue. Many complimentary letters have also come to us, freighted with good wishes for the success of our journal. We are tempted to publish just one as a "specimen;" a liberty which the good nature of the writer will, we trust, pardon. C. E. F.

AIKEN, S. C., Jan. 2, 1884.

*Dr. C. E. Francis:*

DEAR SIR,—Find enclosed postal note for $2.50, for the continuation of my subscription to THE INDEPENDENT PRACTITIONER. It is a journal that improves upon acquaintance. I now enjoy its company, being edified and improved by its worthy articles. I wish it a prosperous New Year. Yours truly,

B. H. TEAGUE.

---

VALUE OF ARTIFICIAL RESPIRATION.

In the *Columbus Medical Journal* for January, may be found an account of a man who swallowed at one time sixteen grains of morphine. No stomach pump could be procured, nor was it possible to induce vomiting. Therefore the whole of the drug was eliminated through the emunctories. Voluntary respiration soon ceased entirely, and for twelve and one-half hours the man was kept alive solely by artificial respiration, when he had so far recovered from the action of the drug that voluntary action was restored, and he finally recovered.

---

REMOVAL.

Ry reference to their advertisement it will be seen that Edward Rowan & Co., formerly of Jersey City, have removed their whole establishment to 196 Third Avenue, New York City. Their facilities for the manufacture and sale of dental goods are largely increased, and they hope and expect that this fact will be fully appreciated by their patrons.

