# A Correction

**Published:** 1884-03

**Authors:** 


### A CORRECTION.

Last month, in an editorial notice of the dissolution of the firm of F. W. Leonard & Co., we perhaps left the impression in the minds of our readers that Mr. Leonard retires from business altogether. This is not the case, as will be seen by a reference to his new advertisement in this number.

